# The expression of FOXP3 in lesions of several forms of leprosy in patients co-infected with HIV

**DOI:** 10.1371/journal.pntd.0009887

**Published:** 2021-11-08

**Authors:** Marília Brasil Xavier, Carla Andréa Avelar Pires, Cláudia Maria de Castro Gomes, Gabriela Fernandes Rodrigues, Débora Pinheiro Xavier, João Augusto Gomes de Souza Monteiro de Brito, Carlos Eduardo Pereira Corbett

**Affiliations:** 1 Research Laboratory in Tropical Dermatology and Endemic Diseases of the Nucleus of Tropical Medicine/Federal University of Pará (NMT/UFPA), Belém, Pará, Brazil; 2 Research Laboratory on Skin diseases of Sanitary Interest, Biological and Health Sciences Center, State University of Pará (UEPA), Belém, Pará, Brazil; 3 Laboratory of Pathology of Infectious Diseases, Department of Pathology, Medical School, São Paulo University, São Paulo, Brazil; Emory University, UNITED STATES

## Abstract

**Background:**

Brazil remains endemic for infection by the human immunodeficiency virus (HIV) and leprosy, having a major impact on public health and the life quality of affected patients. Although the relevance of this co-infection is recognized, several aspects, such as the immune response, are not yet fully understood. The objective of this study was to investigate the expression of FOXP3^+^ Treg cells in leprosy skin lesions and to correlate their clinical forms, laboratory characteristics (CD4, CD8, and CV), and the immune reconstitution syndrome in HIV-leprosy co-infection.

**Methodology/Principal findings:**

An observational, cross-sectional, and analytical study was carried out comparing four groups of patients: those with concomitant diagnosis of leprosy and HIV infection without a leprosy reaction, those with leprosy and HIV co-infection patients with a reverse reaction (RR), those with leprosy without HIV and without reaction, and those with leprosywithout HIV and with RR. The patients were diagnosed at a dermatology outpatient clinic located in Belém, Pará, Brazil, from 2003 to 2017. In the sample studied, there was a positive correlation between FOXP3^+^ cell density and viral load, negative correlation with blood CD4^+^ (not statistically significant), significant positive correlation in CD8 count in patients with leprosy reaction, and positive relationship in patients with IRIS. The density of cells expressing FOXP3 was higher in the BL/LL forms in patients without HIV, although the difference was not statistically significant. However, the cell mean was higher in the TT/BT forms in patients co-infected with leprosy and HIV, showing contradictory results.

**Conclusions/Significance:**

These findings support that higher activity of the HIV may stimulate or result in a higher expression of FOXP3-Tregs and that they may be involved in active immunosuppression observed at the infection site at the tissue level. This supports the need to expand studies on FOXP3^+^ Treg cells in co-infected patients.

## Author summary

Mycobacterium leprae and the human immunodeficiency virus (HIV) cause infectious diseases that have a major impact on public health worldwide. Both are worrisome diseases, with some knowledge gaps not yet fully clarified, regarding their pathogen biology and clinical evolution, especially when patients are infected with them at the same time. FOXP3 (Forkhead box P3) is a transcription factor present in certain regulatory (CD4+ CD25+) Treg cells, which regulates the immune response of the host during intracellular infections, such as tuberculosis and leishmaniasis. This study aimed to elucidate a few of these gaps by exploring the role of FOXP3-positive regulatory T cells (Tregs) on the immune response during leprosy co-infection with HIV, by comparing the response of patients with leprosy based on whether or not they present a HIV and leprosy reaction. The results showed a positive correlation between FoxP3 + cell density and viral load; negative correlation in relation to blood CD4 +. These findings support the conclusion that a higher activity of HIV may stimulate or result in a higher expression of FOXP3-Tregs. However there is a clear need to expand studies on TregFoxP3 + cells in co-infected patients to further elucidate its role in the pathophysiology of these diseases.

## Introduction

*Mycobacterium leprae* and the human immunodeficiency virus (HIV) cause infectious diseases that have a major impact on public health worldwide. Both are worrisome diseases, with some knowledge gaps not yet fully clarified, both in their pathogen biology and clinical evolution, especially when patients are co-infected; Brazil is one of the countries where these endemic diseases overlap [1]. According to data from the Ministry of Health from Brazil, 140,578 new cases of leprosy were diagnosed between 2014 and 2018. In 2018, the presented rate of general detection of new cases per 100 thousand inhabitants was 13.70, classifying Brazil as having a high burden for the disease, second in the world for number of new registered cases. Northern Brazil, located in the eastern Amazon region with a number of diseases and neglected populations, exhibited a general detection rate of 31.95 cases per 100 thousand inhabitants in 2018, twice the national average [2]. There were 300,496 cases of HIV infection (people living with HIV, PLHIV) reported in Brazil from 2007 to June 2019, and about 966,058 cases of AIDS were identified from 1980 to June 2019. The country has registered a decrease in detection rates, except in the North and Northeast regions, which showed an increase of 21.8% and 17.0%, respectively [2]. The increase in cases of AIDS in the Northern region, where the main population of people with leprosy is located, can result in an increase in the prevalence of co-infected individuals, making it opportune to study the co-infection of both diseases in this region.

Leprosy is a disease of the immune spectrum type that is strongly correlated with the extent of its clinical manifestation and polarized immunity against *M*. *leprae* is a critical element in the pathogenesis of the disease. The biased production of Th1 profile cytokines (IFN-γ, IL-2) in the TT spectrum form and Th2 profile cytokines (Il-4, IL-5) in the LL form have been well documented. However, the generation of Th1/Th2-type effector cells alone cannot fully explain the polarized state of immunity. Other subsets of T cells have been identified to play an important role in determining host immunity, including regulatory T cells [3].

Recently, FOXP3-positive regulatory T cells (Tregs) have been characterized as one of the most efficient subsets of suppressor effector T cells, which regulate the immune response caused by the host during intracellular infections, such as tuberculosis and leishmaniasis. The natural regulatory CD4^+^ CD25^+^ Treg cells that express the transcription factor Forkhead box P3 (FOXP3) correspond to a subset of suppressor T cells best characterized thus far. These cells are critical for maintaining self-tolerance and play an important role in a range of clinical conditions, such as autoimmune diseases, transplant rejection reactions, cancer, and infectious diseases. There is no consensus in the literature on the role of Tregs in leprosy. Thus, common knowledge suggests that Tregs can interfere with the Th1 and Th2 response, affecting the immune reaction against mycobacterial infection [4,5].

Since leprosy is a spectral disease, the host immune response transitions between the extremes of the disease, with several obscure areas remaining in the immunology of this disease, mainly in its behavior in association with HIV infection and its various states of immunity. Investigations about the immunopathological peculiarities related to clinical, laboratory, and immune reconstitution syndrome in co-infection with leprosy and HIV/AIDS are important to understand the complexity of these two diseases. Therefore, the objective of this study was to investigate the expression of FOXP3^+^ Treg cells in leprosy skin lesions and to correlate their clinical forms, laboratory characteristics (CD4, CD8, and CV), and the immune reconstitution syndrome in HIV-leprosy co-infection.

## Methods

### Ethics statement

The norms of research with human beings, established by Resolution 466/12 of the National Health Council, were respected, and the recommendations of the ethics committee in research with human beings of the Faculty of Medicine of the University of São Paulo, Brazil, and of the Federal University of Para, Brazil, with approval granted in accordance with opinion number 3.556.477 and 3.377.755, respectively. All studied patients signed a free and informed consent form regarding participation in the research prior to the data collection.

### Study design

An observational, cross-sectional, and analytical study was carried out comparing four groups of patients treated between 2003 and 2017. Group 1 consisted of patients that had a concomitant diagnosis of leprosy and HIV infection without a leprosy reaction (n = 16), Group 2 had co-infected patients with a reverse reaction (RR) (n = 11), Group 3 consisted of patients with leprosy, without both HIV and leprosy reactions (n = 16), and Group 4 consisted of patients with leprosy, without HIV but with a reverse reaction (n = 11). The patients were diagnosed at the sanitary dermatology clinic of the Nucleus of Tropical Medicine (NMT) of the Federal University of Pará (NMT / UFPA), located in the city of Belém, capital of the state of Pará, Brazil.

### Inclusion criteria

Patients aged 18 to 70 years with a diagnosis of leprosy and treated with MDT were included, as recommended by the Ministry of Health [6], classified according to Ridley and Jopling (1962) [7], and diagnosed with HIV/AIDS, according to the Ministry of Health Brazil (2017) [8]. For the characterization of IRIS in co-infected patients, the criteria described by Deps and Lockwood (2010) [9] were used.

Regarding the exclusion criteria, patients who manifested type 2 leprosy reaction, patients with auto-inflammatory or immune-mediated diseases, patients co-infected with HIV who were not on regular use of ART or who were co-infected with other pathogens, such as HTLV, HBV, HCV or *Mycobacterium tuberculosis*, were excluded. In addition, patients who refused to participate in the research or who did not sign the informed consent form were not included.

### Study procedures

#### Clinical and laboratory procedures

Clinical and laboratory procedures were performed at the Sanitary Dermatology Outpatient Clinic and the Clinical Analysis Laboratory of the Center for Tropical Medicine at the Federal University of Pará (UFPA). Immunopathological analyses primarily used immunohistochemical reaction and were carried out in collaboration with the Laboratory of Pathology of Infectious Diseases, department of Pathology, Medical School, São Paulo University, Brazil.

All patients were evaluated by a complete dermato-neurological examination, following the standards of the Ministry of Health (BRASIL, 2016) and underwent complementary tests, such as biopsy with histopathological analysis, to classify the disease according to the Ridley and Jopling criteria and subsequent immunohistochemistry. A search for acid-resistant bacilli in dermal smears was also performed.

#### Histology material collection

A skin biopsy was performed on each patient, collecting the most infiltrated leprosy lesion. Collection was carried out after anti-sepsis and anesthesia (2% lidocaine) at the site, with a punch number 4. The materials were stored in vials containing 10% buffered formaldehyde and then embedded in paraffin. Paraffinized materials were processed as four-micrometer thick histological sections, stained by hematoxylin-eosin and Fite-Faraco stains. For the immunohistochemical study, the sections were mounted on silanized slides and subsequently subjected to immunohistochemical reactions. Patients with reverse reaction had their skin biopsy collected prior to any treatment with corticosteroids.

#### Histopathological analysis

The 4-mm sections were mounted onto slides and stained with hematoxylin and eosin. Ridley and Jopling (1966) criteria were used for the analysis. This classification included two polar types: stable and mutually excluding, tuberculoid (TT) and virchovian (VV) and the dimorphic [dimorphic-tuberculoid, (DT) group; dimorph-dirmorph, (DD); and dimorph-virchovian, (DV)], as well as the indeterminate group, initial phase.

#### Immunohistochemistry technique

To detect the specific marker for FOXP3, a streptavidin-biotin peroxidase immunohistochemical method (SABC, from Streptavidin-Biotin Peroxidase) was used, following the protocol of the Laboratory of Pathology of Infectious Diseases, with modifications. For dewaxing and hydration, the slides were placed at room temperature for 15 minutes. Subsequently, the slides passed through a series of 2 xylol baths, absolute ethyl alcohol, 95%, 80% and 70% ethyl alcohol, and then in distilled water, with two minutes in each passage. Subsequently, the slides were placed for 10 passages in H_2_O_2_ (10 volumes), for three minutes each, to block endogenous peroxidase.

Antigenic recovery was performed with 10 mM citrate buffer pH 6.0 at 96°C in Pascal’s Chamber, for three minutes. To block non-specific connections ("background"), the cuts were treated with a 6% skimmed milk solution (“Molico”, Nestlé) in PBS for 1 hour at 37°C, followed by treatment with 10% fetal bovine serum for 30 minutes at 37°C. The histological sections were then covered by anti-FOXP3 primary antibody (polyclonal, (H: 190): SC-28705, Santa Cruz Biotechnology) diluted 1:250 in bovine serum albumin (BSA) and incubated in an oven at 37°C for 1 hour and then left overnight in a humid chamber at room temperature. Then, the sections were washed in PBS buffer with 0.05% Tween 20 (pH 7.2) and the amplification was performed by the Novolink max polymer system (Novocastra RE7280-K). This was done by incubating the slides with the post-primary conductor at 37°C, then the slides were washed with a PBS-Tween solution and incubated with the polymer for 45 minutes at 37°C, followed by washing the slides with a PBS-Tween solution.

The revelation occurred during incubation with the chromogenic substrate DAB + H_2_O_2_ (3,3´-diaminobenzidine with hydrogen peroxide) for five minutes at room temperature. Then, the slides were washed in running water and distilled water. Counterstaining was performed with Harris hematoxylin for 1 minute, followed by washing in running water and distilled water. After dehydration in an alcohol-xylene gradient, the slides were assembled with coverslips and mounted with a synthetic resin.

For the quantitative analysis of the immunostained cells, the histological sections processed by the immunohistochemistry reaction were analyzed in an optical microscope (Axioskop 2 Plus, Zeiss) coupled to a microcomputer using the program AxioVision 4.3. For this purpose, 10 fields of each histological section (40× objective) were photographed, the brown immunostained cells were quantified, and the average of the quantified fields was divided by the area of the photo (0.03536412 mm^2^) to determine the density of positive cells (cells/mm^2^).

### Patients and methods

The patients were subjected to dermato-neurological examination by a multidisciplinary team composed of dermatologists, infectologists, neurologists, nurses, and physiotherapists. Infection with HIV and AIDS associated with the use of ART were defined according to the guidelines of the MS, including positive serology results from ELISA and WERSTEN BLOTT, followed by flow cytometry for CD4 count in peripheral blood. The criteria for AIDS include a CD4 T lymphocyte count of 200 cells/mL and/or other clinical conditions that define the disease [6]. The diagnosis and classification of leprosy were made according to established clinical criteria, complementary bacilloscopy, and histopathology exams [7,10].

The patients underwent additional testing such as biopsies with histopathologic and dermal smear of lymph to help the classification of the disease according to the Ridley and Jopling criteria [7]. Patients with paucibacillary pole (Tuberculoid Tuberculoid—TT and some Borderline Tuberculoid—BT) were treated for six months with rifampicin and dapsone, and patients with multibacillary leprosy (Boderline Borderline—BB, Borderline Lepromatous—BL, Lepromatous Lepromatous—LL) were treated for 12–24 months with rifampicin, clofazimine, and dapsone. For treatment purposes, borderline tuberculoid patients were classified in the group of paucibacillary after negative smear results, counting the number of lesions with less than five and histopathology compatible with this form. Primary neural patients were classified as PB or MB according to the number of nerve trunks affected. Corticosteroids, in the case of neuritis and type 1 reactions, were administered at a dose of 1–2 mg prednisone per kilogram of body weight per day, being withdrawn slowly according to the clinical parameters presented [6].

### Statistical analysis

The sample was evaluated by descriptive statistics, using measures of central tendency (arithmetic mean), variance (standard deviation), and absolute and relative frequencies. To assess homogeneity between the categories of the groups evaluated, the Chi-square tests of independence, Fisher’s exact test, and G test were used, according to the samples analyzed. Pearson’s Linear Correlation test was used to assess the dependence of the mean cells labeled with FOXP3 on HIV viral load counts and CD4 T cells. Student’s T test was used to assess the difference in means between the groups analyzed. All statistical inferences were calculated using the BioEstat 5.4 and GraphPad Prism 6.0 software, considering a p-value ≤ 0.05 significant.

## Results

The studied sample consisted of 11 co-infected patients with reverse reaction (RR) and 16 without RR (27 co-infected with HIV/Hansen’s disease), in addition to 16 patients with leprosy without HIV and without RR, and 11 patients without HIV and with RR (27 patients with leprosy without HIV), with an absolute total of 54 individuals. The sample showed homogeneity between groups when considering the categories of sex (predominantly male) and age group (young patients, between 15 and 49 years old). As for the origin, there were cases of co-infection that occurred in the interior of the state with a demand for treatment in the reference center located in the capital, while the leprosy cases were all from the metropolitan region of Belém (p = 0.0060) (Table 1).

**Table 1:** Demographic characteristics of leprosy patients with and without HIV coinfection treated at a referral center, Belém—Pará, from 2003 to 2017.

Demographic characteristics	Co-infection group		Hansen’s group		p-value
	n	%	n	%	
Sex					
Male	21	77.78	17	62.96	Chi-square
Female	6	22.22	10	37.04	0.3713
Total	27	100.00	27	100.00	
Age range					
Under 15 years	-	-	2	7.41	G Test
15 to 29 years	7	25.93	6	22.23	0.2126
30 to 39 years	8	29.63	3	11.11	
40 to 49 years	9	33.33	8	29.63	
50 to 59 years	2	7.41	4	14.81	
Over or equal to 60 years	1	3.70	4	14.81	
Total	27	100.00	27	100.00	
Origin					
Metropolitan region	19	70.37	24	88.89	Teste G
State Interior	8	29.63	-	-	0.0060
Ignored*	-	-	3	11.11	
Total	27	100.00	27	100.00	

* Patients with “ignored” results were excluded from the analysis.

Considering the clinical characteristics related to leprosy, the sample presented homogeneity in terms of the therapeutic scheme criteria (slight predominance of the multibacillary scheme) and clinical classification according to the Ridley-Jopling criteria (concentration between the borderline spectra towards the tuberculoid pole), and occurrence of leprosy reaction (both the groups were selected only for patients with reverse reaction) (Table 2).

**Table 2:** Clinical characteristics related to leprosy in patients with and without HIV co-infection treated at a referral center, Belém—Pará, from 2003 to 2017.

Clinical characteristics (leprosy)	Co-infection group		Hansen’s group		p-value
	n	%	n	%	
Therapeutic scheme					
Paucibacillary	12	44.44	10	37.04	Chi-square
Multibacillary	15	55.56	17	62.96	0.7818
Total	27	100.00	27	100.00	
Clinical classification					
TT	6	22.22	6	22.22	G Test
BT	11	40.74	5	18.52	0.3855
BB	8	29.63	13	48.15	
BL	2	7.41	2	7.41	
LL	-	-	1	3.70	
Total	27	100.00	27	100.00	
Occurrence of leprosy reaction (reverse reaction)					
Yes	11	40.74	11	40.74	Fisher Exact
No	16	59.26	16	59.26	1.0000
Total	27	100.00	27	100.00	

* TT: Polar tuberculoid; BT: tuberculoid Boderline; BB: Boderline borderline; BL: lepromatous Boderline; LL: Polar lepromatous.

Among the co-infected patients, there was a predominance of those manifesting with AIDS and using antiretroviral therapy, despite the presence of a leprosy reaction. Most of the patients were in the absence of IRIS (Table 3).

**Table 3:** Clinical characteristics related to HIV in patients with and without leprosy reaction treated at a referral center, Belém—Pará, from 2003 to 2017.

**Clinical characteristics (HIV)**	**With reaction**		**Without reaction**		**p-value**
	**n**	**%**	**n**	**%**	
Use of Antiretroviral Therapy					
Yes	10	90.91	13	81.25	G Test
No	1	9.09	3	18.75	0.7228
Total	11	100	16	100	
AIDS					
Yes	10	90.91	11	68.75	G Test
No	1	9.09	5	31.25	0.3625
Total	11	100	16	100	
Immune Reconstitution Syndrome					
Yes	5	45.45	4	25	G Test
No	6	54.55	12	75	0.4902
Total	11	100	16	100	

**Fig 1:**
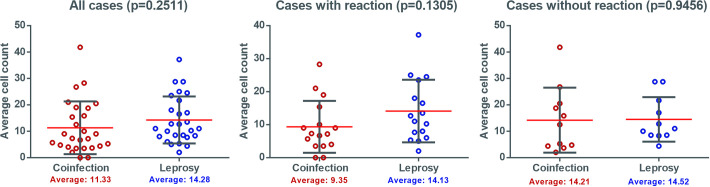
Density of cells expressing FOXP3 in lesions of patients with leprosy with and without HIV co-infection.

There was no difference between the means of cells marked by FOXP3, when considering the reaction state, in almost all cases. An average count of between 10 and 20 cells were marked in the analyzed samples (Fig 1).

**Fig 2:**
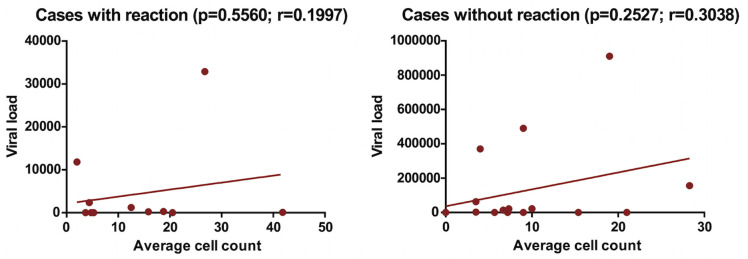
Cells marked with FOXP3 and viral load among HIV/leprosy co-infected patients, according to the presence of reverse reaction.

**Fig 3:**
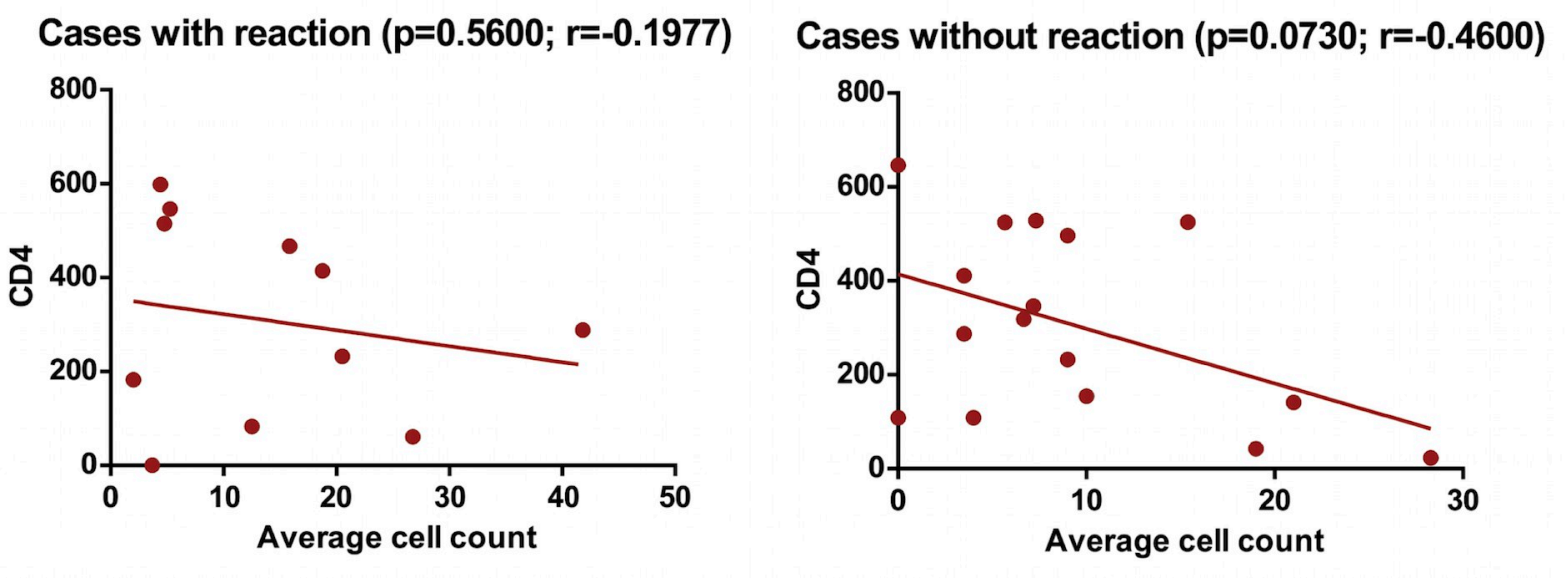
Cells marked with FOXP3 and CD4 T cells among patients with HIV/leprosy coinfection, according to the presence of reverse reaction.

**Fig 4:**
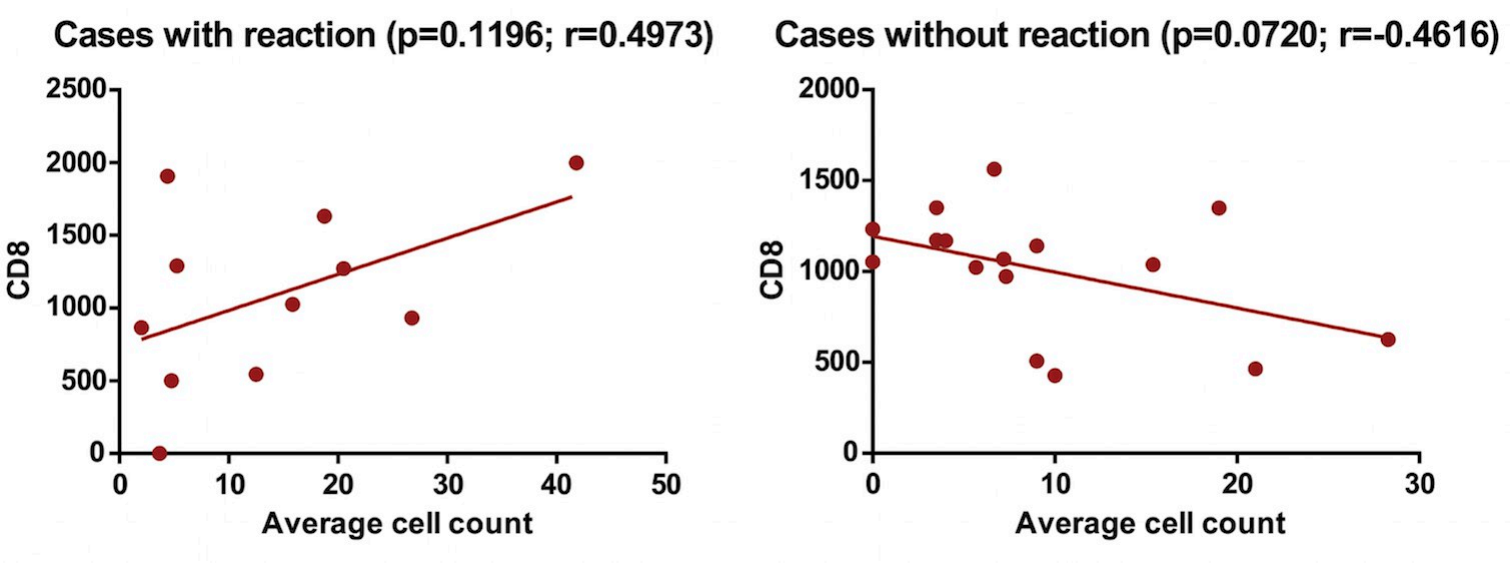
Correlation of cells marked with FOXP3 and CD8 T cells among patients with HIV and with leprosy coinfection, according to the presence of reverse reaction.

There was a positive correlation between cell count and viral load, while there was a negative correlation between cell count and CD4, considering the presence of a leprosy reaction. When the CD8 count was evaluated, a positive correlation was noticeable in cases with reaction while there was a negative correlation in cases without reaction (Figs 2, 3 and 4).

**Fig 5:**
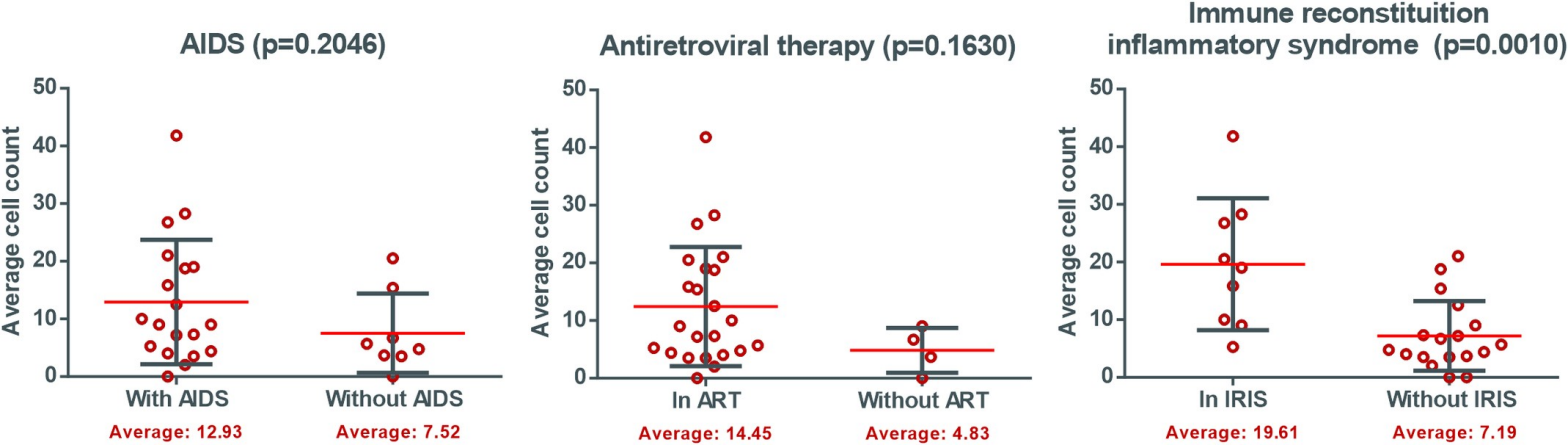
Density of cells expressing FOXP3 in leprosy patients with HIV co-infection according to the presence of AIDS, ART and IRIS. *Antiretroviral therapy (ART), Immune Reconstitution Syndrome (IRIS),

As for HIV-related characteristics, there was a significant difference (p = 0.0010) regarding the presence of IRIS, with a higher mean and dispersion of samples in patients on IRIS, as opposed to a lower average of cells marked in the absence of IRIS (Fig 5).

**Fig 6:**
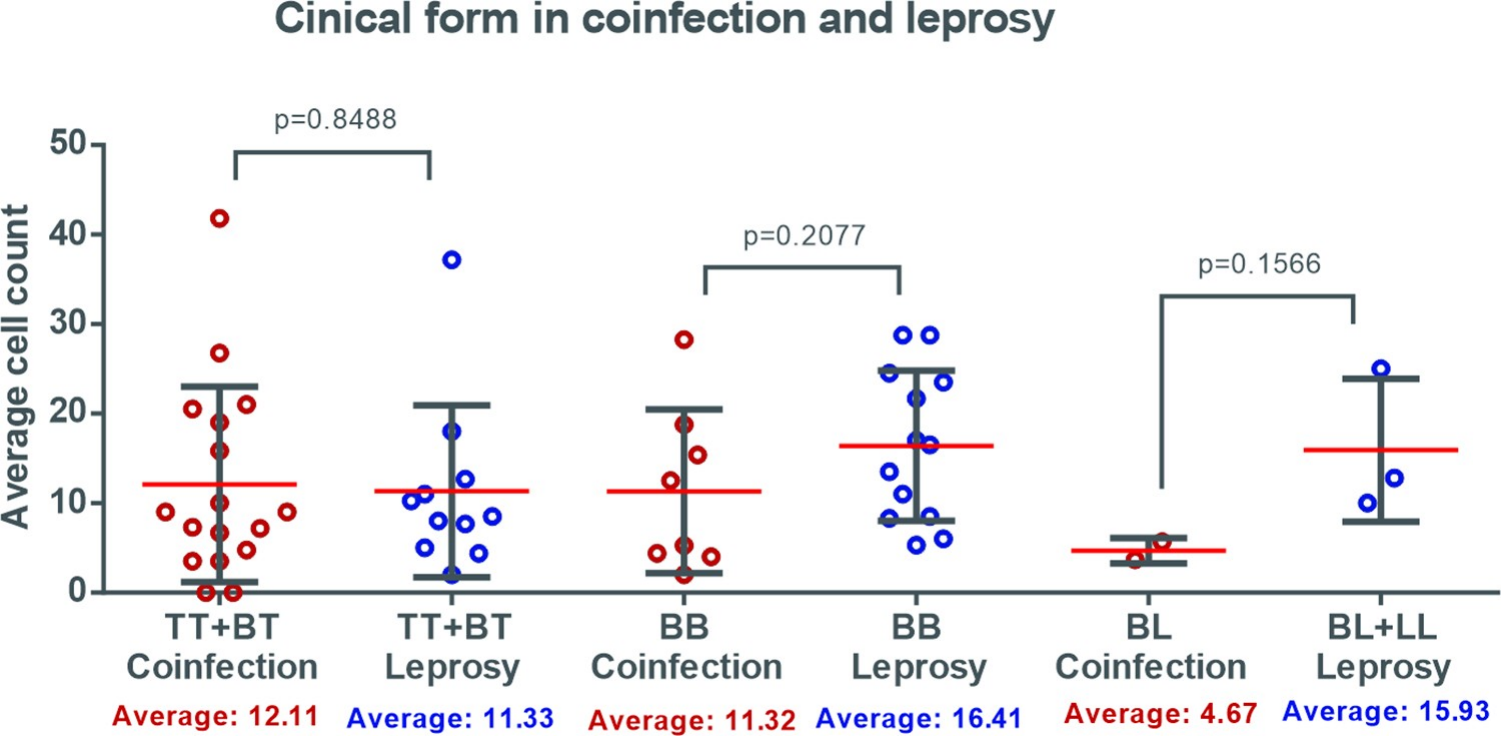
Density of cells expressing FOXP3 in patients with leprosy with and without HIV co-infection, according to clinical form. * (comparison between groups with isolated borderline borderline). *Clinical forms: borderline borderline (BB), borderline tuberculoid (BT), tuberculoid (TT), borderline lepromatous (BL) and lepromatous (LL).

Grouping the clinical forms, there was a higher average of cells among patients co-infected with borderline forms towards the tuberculoid pole. When the borderline form was isolated, it matches the tuberculoid + tuberculoid borderline forms. When the equivalent categories between groups were evaluated, there was an equivalence between the forms towards the tuberculoid pole and higher averages in the leprosy group in the other evaluations, mainly considering the forms towards the lepromatous pole, although they were not statistically significant (Fig 6).

**Fig 7:**
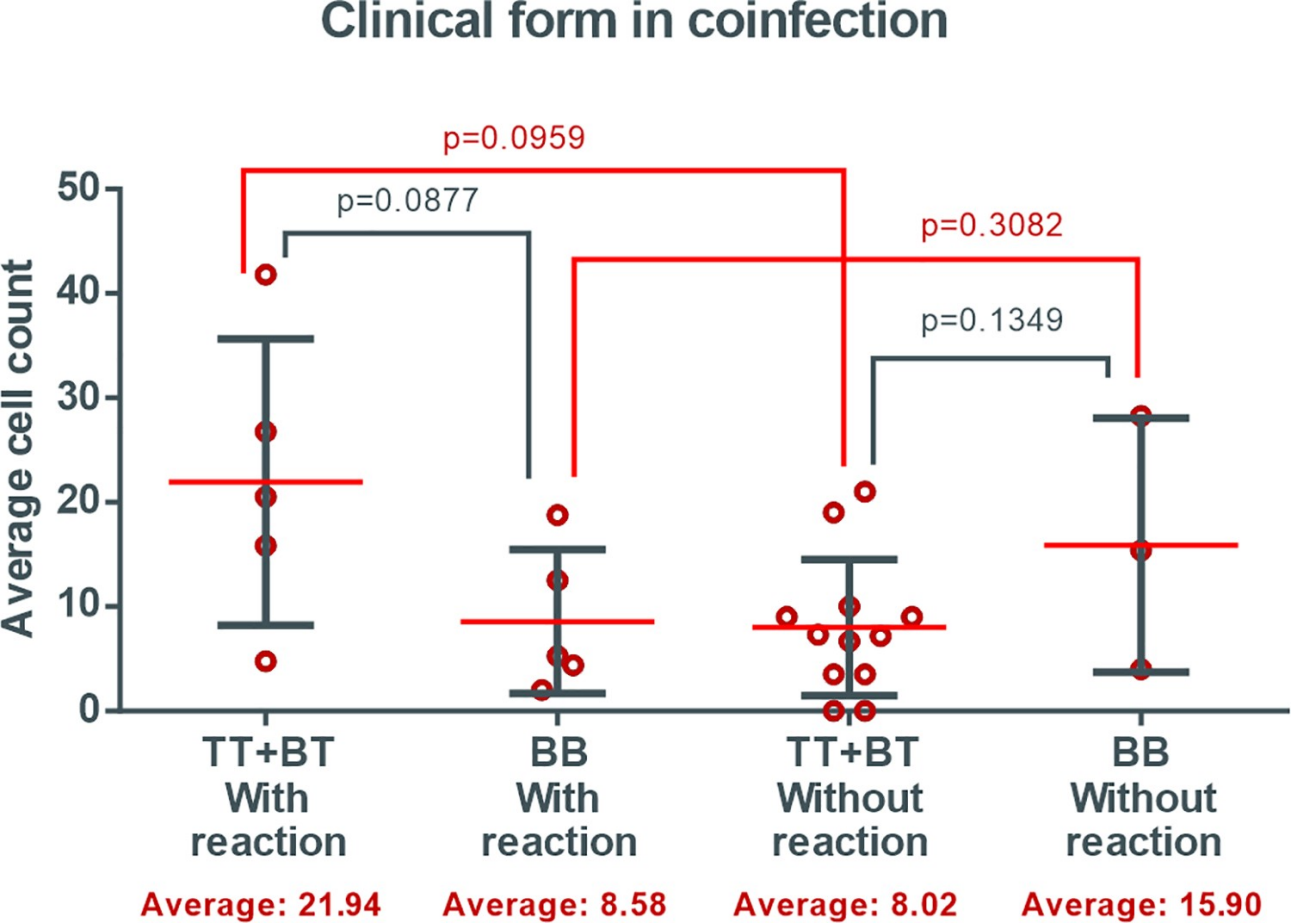
Density of cells expressing FOXP3 in patients with leprosy co-infected with HIV, according to clinical form* and presence of reverse reaction. *Clinical forms: borderline borderline (BB), borderline tuberculoid (BT) and tuberculoid (TT).

**Fig 8:**
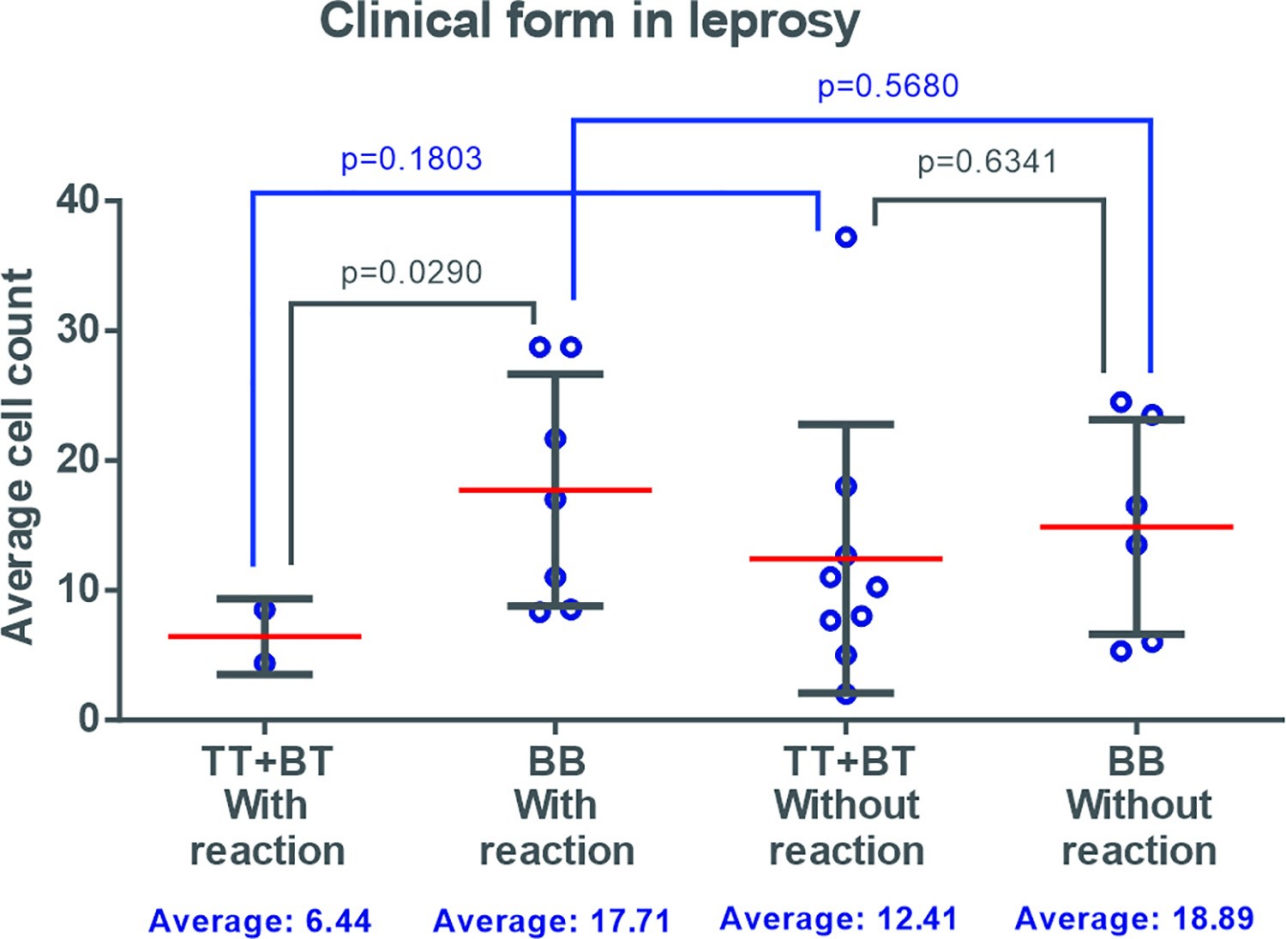
Density of cells expressing FOXP3 in patients with leprosy, according to clinical form* and the presence of reverse reaction. *Clinical forms: borderline borderline (BB), borderline tuberculoid (BT) and tuberculoid (TT).

There was no statistical difference in patients with co-infection according to the isolated clinical forms, considering the presence of a leprosy reaction, with the highest mean present in the borderline group of tuberculosis in reaction (Fig 7). In the leprosy group, there was a significant difference between patients with reaction (p = 0.0290), with the highest averages in the group present in borderline borderline patients in reaction (Fig 8). When both groups were evaluated, the highest mean and perceived dispersion came from the borderline tuberculosis clinic co-infected group among the patients in reaction. In patients with an absence of a leprosy reaction, there were similarities in the mean cell counts for the groups evaluated.

## Discussion

The demographic results demonstrate agreement with the literature, pointing to the most common co-infection cohort: men aged between 15 and 49 years [11]. There was no significant difference in the clinical forms expressed between the groups studied. A predominance of the clinical form borderline tuberculoid (BT) was observed in co-infected patients (40.74%), and the borderline borderline (BB) form was more frequent in the group of patients without HIV (48.15%), confirming what has been reported in the literature [12–17]. Case series reports and some co-infected patient cohorts point out that diseases appear to follow their natural histories with little interference [12,13,18]. The initial expectations were that there would be an increase in severe cases of multibacillary leprosy due to an increase in AIDS cases, which was not evidenced in studies carried out in Africa and Brazil [19].

The frequency of reaction states, which are phenomena of acute hypersensitivity that occur in the presence of *Mycobacterium leprae* antigens and result from an immunological process, are not yet fully understood. However, they are already characterized by an increase in pro-inflammatory cytokines, especially interleukins: IL-1β, IL-12, IL-2, IL- 4, IL-6, IL-10, among others, in addition to immunocomplexes [20]. It was not the focus of this study, as the same number of patients who had a reverse reaction between groups of co-infected and non-co-infected were used to perform the cell count and subsequent comparison. We opted to exclude patients who had a type 2 leprosy reaction.

Even though leprosy and HIV are endemic diseases with high prevalence in the northern region of Brazil where the study was carried out, their co-infection is still a rare event [1,2]. In the present study, we chose to exclude all patients with autoinflammatory and autoimmune comorbidities, as well as patients with other infectious diseases, in order to standardize the sample, thus avoiding variables that could eventually modify the immune response and generate bias, which further reduced the number of patients, reaching a total of 54.

Chronic HIV infection is associated with several co-infections that contribute to immune activation in several perceptible contexts, through the immune reconstitution syndrome, after the onset of ART. Some studies indicate that HIV/leprosy co-infection may also be directly related to the immunological improvement that the introduction of antiretroviral therapy (ART) provides, and, therefore, develop milder and more limited forms of the disease. It is believed that, if they did not have HIV infection, these patients might not get sick from leprosy [19,21–23]. This association is strongly suggested by some case series or case reports that demonstrate the temporal relationship between the onset of leprosy symptoms, of HIV disease, and of ART [12]. Although not the most common, there are also cases which described patients manifesting leprosy without having AIDS, only HIV infection, and still not using ART. In one study, 22% of patients had leprosy with RR before patients started using ART [12], which was also observed in two co-infection cases described by Trindade et al. (2006) [24],

Histologically, leprosy is characterized by a spectrum of different granulomatous lesions of the skin, reflecting patients’ immune response to *Mycobacterium leprae*. The immunopathological characteristics and aspects of leprosy granuloma in co-infection with HIV also seem to be conserved, according to what was described in the literature [25,26]. Another exciting aspect that has been demonstrated is that, in the emergence of leprosy, clinical forms and cure with standard polychemotherapy (MDT) are not related to CD4 levels or viral load [12–14,25,27].

Biological systems are subject to complex immune regulatory controls, and one of the control points of the immune response is established when the normal immune response is initiated. Therefore, another mechanism must be put in place to control the magnitude of this response and to end it at the right time. This regulation should contribute to limiting the clonal expansion and the activity of inflammatory cells. Natural regulatory T cells (Treg) are involved in this regulatory mechanism, constitutively expressing CD25. FOXP3 (Forkhead box P3) is the most specific marker available for this mechanism so far [28].

Treg cells can be qualitatively or quantitatively altered in human skin diseases, suggesting their role in the pathophysiology of these diseases. Natural Tregs (CD25^+^FOXP3^+^ cells) are intended to maintain tolerance, suppressing the function of autoreactive T cells in different skin diseases, and have been studied for their involvement in various diseases, including leprosy [28]. Of the various mechanisms proposed by the host to regulate immune damage caused by excessively robust immune responses, Treg cells are believed to play a critical role in regulating inflammatory responses by mediating the main components that facilitate immune suppression. Mediators of suppression induced by Treg cells include the inhibitory cytokines IL-10 and TGF-β. Tregs have also been shown to control excessive inflammatory responses against pathogens. However, strict control of effector T cell responses by Tregs can favor infection and promote the persistence of pathogens [3,29,30].

There is no consensus in the literature on the role of Tregs in leprosy. Common knowledge suggests that Tregs can alter the Th1 and Th2 responses, interfering with the immune response against mycobacterial infections [4]. The divergence in findings is seen in several reports. Attia (2014) [31] reported that large quantities of Tregs are present in patients with the TT clinical form, suggesting that Treg activity may be beneficial for patients with leprosy. In contrast, Pallermo et al (2012) [32] showed a larger population of Tregs in the lepromatous clinical form, inferring that they may also have a pathogenic role in patients at the lepromatous pole. The present study showed a high mean cell number of FOXP3 in BL/LL forms in patients without HIV, corroborating the findings of Palermo (2012) [32]. However, the mean density values of Tregs were greater in the clinical forms of TT/BT coinfection than in other clinical forms, which may be related to the fact that these clinical forms were predominant in patients who presented with IRIS, as was the case in 4 BT and 3 TT patients and in no BL or LL patients.

Regarding the presence of an RR, the distribution of mean cell numbers of FOXP3 was demonstrated to be homogeneous with higher mean cell values in the BB clinical form, both in patients without RR and in those who presented the reaction episode. Massone, et al. (2010) [30] observed a significant difference in FOXP3 expression in patients with leprosy RR (BT-RR and BB-RR), when compared with the non-reaction forms (BL, BT, LL, TT), which may suggest a positive role for Tregs in leprosy.

Massone (2011) [33] studied FOXP3+ in patients coinfected with HIV-leprosy in a reduced sample of 14 patients, with five individuals in RR and four in IRIS, and observed that FOXP3+ cells were detected in all clinical forms of leprosy in an average of 3%–4% of cells per lymphocytic infiltrate, similar to results in cases of HIV-negative leprosy seen previously by the same author. In the present study, a mean count of between 10 and 20 cells marked in the analyzed sample was observed, and this average was slightly higher in BT patients with RR, although there was no significant difference. The increase in Treg cells density during RR suggests a suppressive role in controlling the exacerbated cellular immune response, which could cause considerable damage to the tissues and nerves [34].

In this study, although only the expression of FOXP3 was investigated as a marker for Tregs, which can be considered a limitation, the detection of the nuclear FOXP3 expression has been widely used as an accurate indicator of the role of Tregs. To date, it is known that in co-infected patients, the frequency of FOXP3+ cells does not seem to be influenced by the clinical type of leprosy, the CD4+ T cell count in the peripheral blood, or the HIV viral load [33]. However, in this study, there was a positive correlation to viral load, negative correlation to blood CD4^+^, and a noticeable positive correlation with the CD8 count in the patients with RR. Recently, CD8^+^ FOXP3^+^ Tregs were identified in granulomas of tuberculous lymphadenitis, suggesting that they may be involved in active immunosuppression observed at the infection site [35].

AIDS status and ART use were not associated with the average expression levels of Treg cells; however, patients who were in the IRIS group had a significantly higher average expression level (p = 0.0010). There are only a few studies on Tregs in patients co-infected with HIV and leprosy; although Treg cells have been defined as a key population during HIV-1 infection, longitudinal studies investigating the frequency of Tregs in HIV-1 infection have shown divergent results. Consequently, the role of Tregs in the pathogenesis of HIV-1 also remains uncertain [36].

The first case of IRIS manifested with RR opening a clinical picture of leprosy was published by Lawn et al (2003), and since then, several similar cases have been described [37]. The largest cohort published in Brazil on this association (RR and IRIS) was written by Menezes et al. (2011) [37]. Massone et al. (2010) [30] observed a numerical increase in regulatory T lymphocytes (LTreg), but with functional impairments in patients with IRIS. Several assumptions are involved in this process, and it may be that this immunological dysregulation, which culminates in the rapid increase in CD4^+^ cells and a decrease in the viral load, is a possible explanation for these patients with HIV to manifest, for the first time, the clinical lesions of leprosy with symptoms of intense reactions, after the use of ART (known as “unmasked” IRIS), or in others already diagnosed with leprosy, to present only the inflammatory reaction also after the use of ART (known as “paradoxical” IRS). [38,39]. However, according to Talhari et al. (2008) [40] and Ustianowski, Lawn, and Lockwood (2006) [41], it remains unclear whether IRIS triggers the normal presentation of leprosy or whether the natural history of leprosy was accelerated by IRIS in these cases. There are still many gaps on the underlying mechanisms of IRIS, as well as the immune response to the bacillus that causes leprosy.

In summary, data from several studies indicate that Treg cells are essential modulators of immunity against different pathogens. Knowing and understanding all the pathophysiological mechanisms involved in both diseases that are major obstacles to public health is essential for new discoveries, which may enable, in the future, new ways of acting on this matter. It was suggested that the frequency of FOXP3^+^ cells in the skin was not influenced by the clinical type of leprosy, CD4^+^ T cell count, or HIV viral load. However, the present study identified a positive correlation between FOXP3^+^ cell density and viral load, negative correlation with blood CD4^+^, although not statistically significant, significant positive correlation in CD8 count in patients with leprosy reaction, positive relationship in patients with IRIS, and higher average density values of Treg cells in the clinical forms of TT/BT co-infection than in other clinical forms. These findings support that higher activity of the HIV may stimulate or result in a higher expression of FOXP3-Tregs and that they may be involved in active immunosuppression observed at the infection site. However, study limitations include a small sample and the use of only FOXP3^+^ cells to investigate the role of Tregs. Longitudinal studies investigating the frequency and function of Treg in HIV/leprosy co-infection need to be encouraged to better elucidate the immunological mechanisms involved.
